# Rupture Test: A New Method for Evaluating Maize (*Zea mays*) Seed Vigour

**DOI:** 10.3390/plants13131847

**Published:** 2024-07-04

**Authors:** Heqin Li, Haiwang Yue, Miaomiao Lu, Ru Jia, Xuwen Jiang

**Affiliations:** 1 College of Agronomy, Qingdao Agricultural University, Qingdao 266109, China; hqliaau@163.com (H.L.); lumiaomiao322@163.com (M.L.); ru.jia@stu.qau.edu.cn (R.J.); 2 Dryland Farming Institute, Hebei Academy of Agriculture and Forestry Sciences, Hengshui 053000, China; yanjiu1982@163.com

**Keywords:** seed vigour, germination, pericarp–testa rupture, coleorhiza rupture, seed biomechanics, maize

## Abstract

To explore the application of seed germination biomechanical event(s) in seed vigour tests, a new procedure for the evaluation of maize seed vigour tests based on pericarp–testa rupture (PR) and coleorhiza rupture (CR) during seed germination was developed. Twenty–four lots of hybrid maize were used to determine the feasibility of the rupture test (RT) as a seed vigour test in *Zea mays*. The results showed that the physiological quality pattern of 24 maize seed lots assessed through RT was similar to that obtained through analysis with other seed test methods. Correlation and regression analyses revealed that the percentage of CR and percentage of PR + CR at “15 ± 0.5 °C for 120 h ± 1 h” and “20 ± 0.5 °C for 72 h ± 15 min” exhibited positive correlations with the field seedling emergence data (*p* < 0.01). Hence, the proposed method (the rupture test) is cogent and effective, thus providing an important reference for more crops to select for seed germination event(s) and establishing corresponding new methods for seed vigour tests in the future.

## 1. Introduction

Seed physiological quality directly affects field emergence and crop production. By definition, seed vigour is defined as those seed properties that determine the potential for rapid, uniform emergence and development of normal seedlings under a wide range of field conditions [1]. Germination vigour is driven by the ability of the plant embryo to begin metabolic activity in a coordinated and orderly manner [2]. The quiescent, dry seed absorbs water to initiate seed germination, which is followed by the embryonic axis lengthening [3]. It usually results in the radicle emerging and the seed coat rupturing and is regarded as the end of germination [4]. According to a mechanical perspective, the germination process should be visible as a communication and interaction between two opposing powers: the development capability of the embryo and the limiting power of the seed covering layers [5]. Based on differences in the composition of the cell wall and water absorption capacity, different seed tissues can be regarded as composite materials with different dynamic properties. During seed germination, the biomechanical mechanism of embryonic cell growth depends on irreversible loosening of the cell wall, trailed by water take–up because of decreased turgor pressure, leading to embryonic elongation and eventual radicle (embryonic root) emergence [5,6]. A large number of studies on physiological, biochemical, and molecular regulation of seed germination and vigour have been summarised in many literature reviews [2,4,5,6,7,8,9], and relevant research results have been the physiological basis for explaining the principles behind standard germination and vigour tests (electrolyte leakage, cold test, ageing, germination rate, and seedling size) [10]. However, it is rare to apply the results of seed germination biomechanics as the principle of seed vigour tests [5,11]. The basic objective of seed vigour testing is to precisely identify important differences in physiological potential among commercially valuable seed lots [12]. Therefore, simple, rapid, and accurate seed vigour testing methods based on the biomechanical principle of seed germination are worth exploring and studying.

Maize (*Zea mays* L.) is an important grain-forage and energy crop worldwide [13]. The production of high–quality hybrid maize seeds is one of the foundations of successful agriculture and can comprehensively enhance the development level of modern agriculture. In a maize caryopsis, the embryonic shoot is contained by the coleoptile, and the primary root (embryonic root) is contained by the coleorhiza [14]. As a kind of biomaterial, plant seeds have complex structures and exhibit complex mechanical behaviour in response to external loading [15,16]. Cellulose, hemicellulose, pectin, lignin, and protein are important components of the plant cell wall. This rigid structure, together with the osmotic properties of protoplasts, controls the mechanical properties of cells, tissues, and organs [17,18,19,20]. Combining the perspectives of biologists and material scientists on structure and mechanics is a timely approach to advance our understanding of plants and provide new insights on biomaterials.

**Figure 1:**
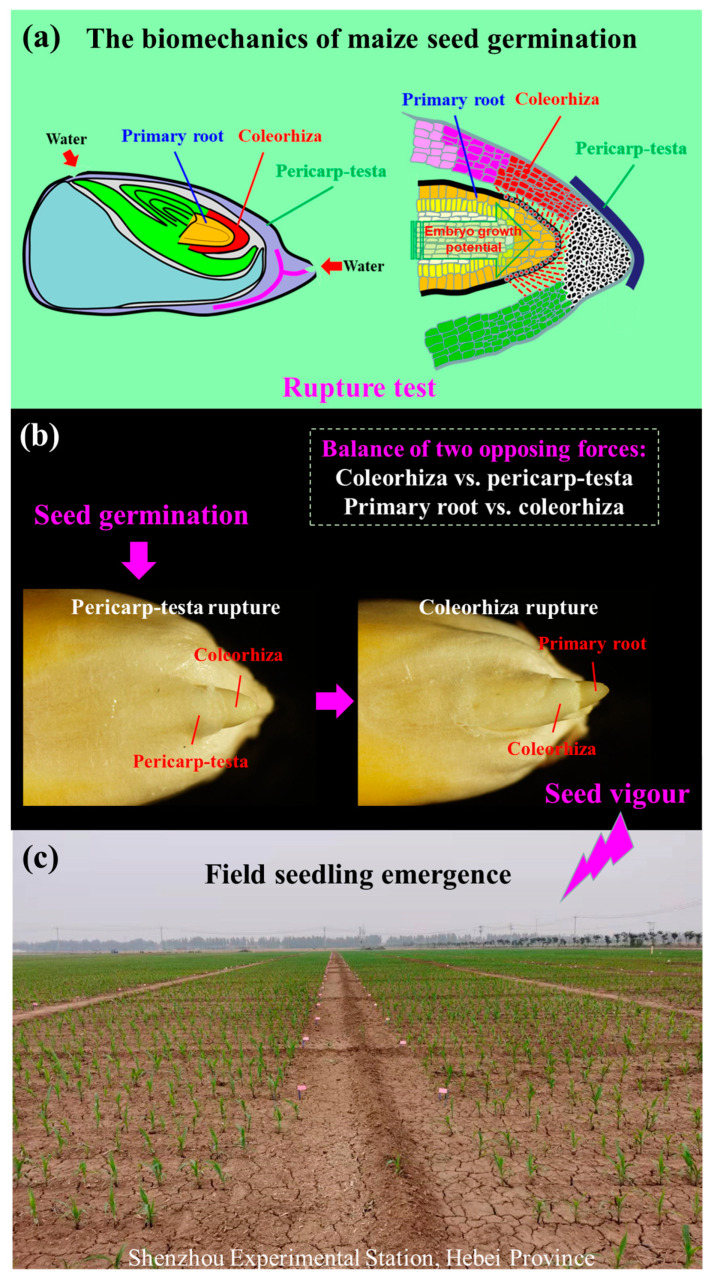
Rupture test for *Zea mays*. (**a**) Schematic diagram of the rupture test. (**b**) Pericarp–testa rupture and coleorhiza rupture. (**c**) Field seedling emergence test.

Two qualitative changes in biomechanical events in accordance with the characteristics of maize seed germination were proposed: (i) pericarp–testa rupture (PR): the balance of forces between the coleorhiza and the pericarp–testa is disrupted, and the coleorhiza protrudes from the pericarp–testa; and (ii) coleorhiza rupture (CR): the balance of forces between the primary root and the coleorhiza is disrupted, and the primary root protrudes from the coleorhiza (Figure 1a,b). Therefore, it is worth studying whether these two biomechanical events during maize seed germination can be used to evaluate seed lot vigour (Figure 1c).

Seed ageing leads to the loss of seed vigour, increases the lag period before primary root emergence, and negatively affects field seedling emergence [21]; it also increases the lag period before PR and CR in maize [15]. In the present study, the pericarp–testa rupture percentage (PRP), coleorhiza rupture percentage (CRP), pericarp–testa rupture + coleorhiza rupture percentage (PRCRP), germination first count (GFC), germination percentage of the cold test, germination percentage of the accelerated ageing test, primary root emergence (PRE), field seedling emergence, and other test results of twenty–four maize seed lots were compared. The purpose of this study was to provide experimental evidence showing whether the rupture test can be used as a new method of maize seed vigour testing and to provide reference for the study of more related new biomechanical evaluation methods of seed vigour. Note: For a list of all abbreviations, see Figure S1.

## 2. Results

### 2.1. The Seed Length (SL), Seed Width (SW), 1000–Seed Weight (TSW), and Seed Water Content (SWC)

The SL and SW of the 24 maize seed lots of the eight cultivars ranged from 10.51 to 12.14 mm and from 7.87 to 9.80 mm, respectively. The difference in SL and SW between different seed lots of the same variety was not significant. The four seed lots of the American cultivars were slightly smaller than those of the four Chinese cultivars. The TSW of the 24 maize seed lots of eight cultivars ranged from 295.81 to 382.92 g (Tables S1 and S2). The SWC of the 24 seed lots ranged from 11.31 to 11.72%, which was below the safe seed storage water content (<13%) (Tables S1 and S2).

### 2.2. Germination Test and Seedling Growth Test

**Figure 2:**
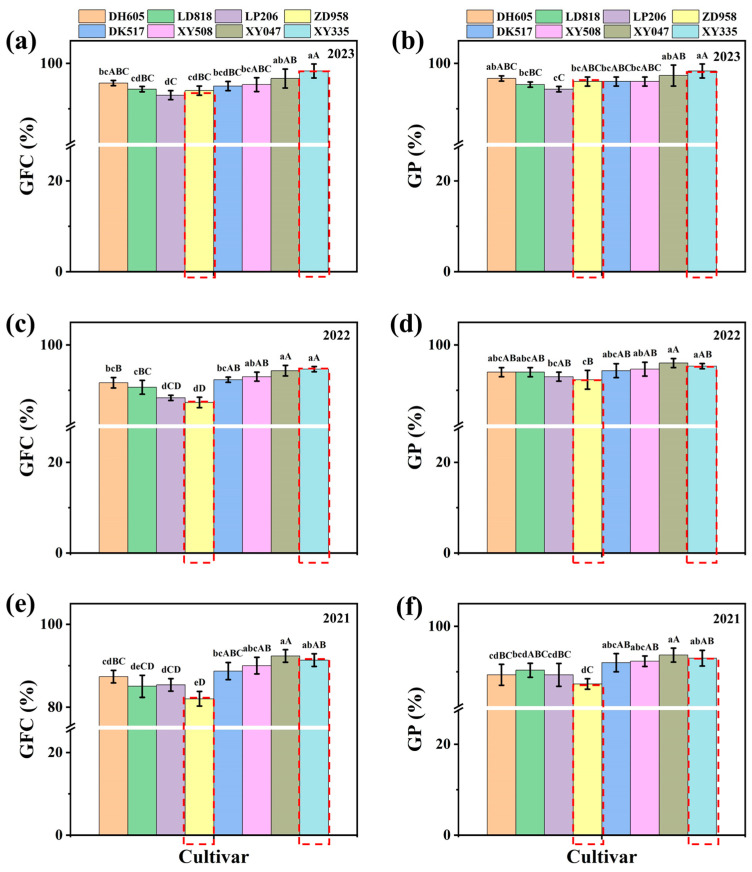
Comparative analysis of the germination first count (GFC) and germination percentage (GP) of different maize cultivars. (**a**) GFC of samples in 2023. (**b**) GP of samples in 2023. (**c**) GFC of samples in 2022. (**d**) GP of samples in 2022. (**e**) GFC of samples in 2021. (**f**) GP of samples in 2021. Different lowercase letters mean a significant difference (*p* < 0.05), and different capital letters mean an extremely significant difference (*p* < 0.01). The red dashed boxes represent the representative Chinese hybrid maize cultivar ZD958 and the representative American hybrid maize cultivar XY335.

According to a comparative analysis of the physiological indices of ten seeds from the 24 maize hybrid seed lots of eight cultivars, the indices of three seed lots of the same cultivar all exhibited the same trend in change: 2023 seed lot > 2022 seed lot > 2021 seed lot; the differences in the vigour indices (such as the GI and VI) among the three seed lots of the same cultivar were significant (*p* < 0.05) or extremely significant (*p* < 0.01). Seed deterioration has become recognised as the major reason for the decline of seed vigour [6]. The vigour of the eight hybrid maize cultivars decreased with increasing storage time, but the degree of deterioration differed among the different maize cultivars (Table S3). According to the GFC and GP, there were differences among the eight maize variety lots in the same year. The degree of deterioration of the four American varieties of seed lots was significantly lower than that of the four Chinese varieties. Among the eight varieties, ZD958 and LD818 had the greatest degree of deterioration, while XY335 and XY047 had the lowest degree of deterioration. According to the GFC, for ZD958 and XY335, the seed vigour of the ZD958 seed lot was extremely significantly (*p* < 0.01) lower than that of the XY335 seed lot in the same year, which may be related to the genetic defect of ZD958 in seed vigour (Figure 2).

### 2.3. Cold Test, Accelerated Aging Test, and Primary–Root Emergence Test

The results of CT, AAT, and PRET showed that there were differences in the seed vigour of the same cultivar in different seed production years. The change trend in each index of the three seed lots of the same cultivar was the same: 2023 seed lot > 2022 seed lot > 2021 seed lot (Table 1). The two indices of PRET (PRET–13 °C, 144 h; PRET–20 °C, 66 h) also conformed to the above trend. The results of four seed vigour indices of the eight seed lots in 2021 were comprehensively analysed and compared, and the seed vigour of ZD958-2021 was the lowest, and its four vigour indices (CT, AAT, PRET–13 °C, 144 h, PRET–20 °C, 66 h) were 84%, 70.33%, 65.33%, and 80.67%, respectively. The highest seed vigour was detected in XY335–2021, with values of 88%, 71%, 72.67%, and 88.33%, respectively (Table 1).

### 2.4. Field Seedling Emergence

Seed ageing causes the loss of seed vigour, which results in low seedling emergence in the field [2]. Three field seedling emergence indices (FSE–J, FSE–L, and FSE–S) of 24 seed lots of 8 hybrid maize cultivars were compared and analysed, and the FSEs of three seed lots of the same cultivar were 2023 seed lot > 2022 seed lot > 2021 seed lot (Table 2). The seed vigour of ZD958–2021 was the lowest, and the FSE–J, FSE–L, and FSE–S were 78%, 79.33%, and 79.67%, respectively. Compared with those of ZD958-2021, the seed vigour of XY335–2021 was greater, and the FSE–J, FSE–L, and FSE–S of XY335–2021 were 86%, 84.33%, and 86.33%, respectively (Table 2).

### 2.5. Comparative Analysis of PRP, CRP, and PRCRP

The curves of PRP, CRP, and PRCRP with the extension of germination time for 24 seed lots at four germination temperatures (13, 15, 20, 25 °C) were similar. With increasing germination time, the change in the PRP curve showed a gradual increase and then a gradual decrease. The curves of CRP and PRCRP were similar; both increased continuously and then tended to stabilise during germination (Figures S2 and S3). Deteriorated seeds have a longer lag period than fresh seeds, as indicated by the longer time needed for PR and CR. Compared with that at 25 °C, the lower temperature decreased the speed of germination and increased the difference between seed lots with different vigour levels. However, when the germination temperature is low, it takes a long time for low–vigour seed lots (such as ZD958–2021) to complete the two germination biomechanical events of PR and CR (Figures S2 and S3). The results of the correlation analysis of PRP, CRP, and PRCRP with FSEs (FSE–J, FSE–L, and FSE–S) showed that PRP–13 °C, 96 h, PRP–15 °C, 48 h, PRP–15 °C, 72 h, PRP–20 °C, 48 h, PRP–25 °C, 24 h, and PRP–25 °C, 48 h were significantly or extremely significantly correlated with FSEs (FSE–J, FSE–L, and FSE–S), and the CRP and PRCRP at 72–192 h of 13 ± 0.5 °C, 72–192 h of 15 ± 0.5 °C, 48–108 h of 20 ± 0.5 °C, and 48–168 h of 25 ± 0.5 °C were significantly or extremely significantly correlated with FSEs (FSE–J, FSE–L, and FSE–S) (Table S4).

### 2.6. Selection of the Evaluation Indices for the Rupture Test

**Figure 3:**
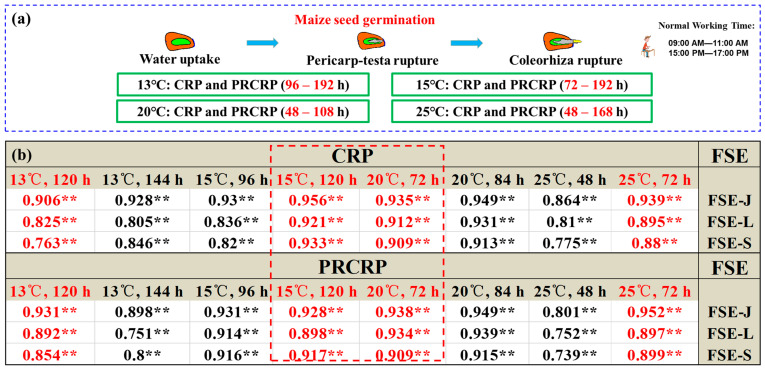
Selection and determination of the evaluation indices for the rupture test. (**a**) The principle of selecting indices for evaluating seed vigour by rupture test. (**b**) Relationships between the seed lot vigour indices in the rupture test and FSEs (FSE–J, FSE–L, and FSE–S). For the complete list of abbreviations, go to “Figure S1”. ** indicates significance at *p* < 0.01.

The evaluation time intervals of PRP, CRP, and PRCRP at the four germination temperatures were as follows: 96–192 h at 13 ± 0.5 °C, 72–192 h at 15 ± 0.5 °C, 48–108 h at 20 ± 0.5 °C, and 48–168 h at 25 ± 0.5 °C (Figure 3, Figures S4 and S5 and Table S4). The test should be set up at a convenient time of day for subsequent index evaluation. On the one hand, it is best to set the starting and ending time points of the rupture test (RT) within a reasonable time interval, such as the normal working time, which is usually from 9:00 a.m. to 11:00 a.m. and from 15:00 p.m. to 17:00 p.m.; on the other hand, the maize seed vigour evaluation index selected at RT should be able to quickly and accurately reflect the potential performance of a seed lot. Finally, high temperatures increased the germination speed of seed lots and reduced the seed vigour difference between seed lots; in contrast, seed lots that germinate too slowly at very low temperatures can affect the efficiency of the rupture test. Therefore, considering all factors, the appropriate evaluation index system for the maize rupture test was as follows (Figure 3):

(i) At 15 ± 0.5 °C, the recommended indices are “CRP, 120 h ± 1 h” and “PRCRP, 120 h ± 1 h”. For example, if the test is set up at 10:00 a.m., the count will occur at 10:00 a.m. five days later (count at 5 days ± 1 h).
(ii) At 20 ± 0.5 °C, the recommended indices are “CRP, 72 h ± 15 min” and “PRCRP, 72 h ± 15 min”. For example, if the test is set up at 10:00 a.m., the count will occur at 10:00 a.m. three days later (count at 3 days ± 15 min).

The CRP and PRCRP can be obtained at one time, and it will be more accurate to evaluate the vigour of a seed lot by using these two indices.

### 2.7. Relationship between Maize Seed Vigour Indices

There were positive correlations for CRP–15 °C, 120 h (r = 0.956, 0.921, 0.933, respectively; *p* < 0.01), PRCRP–15 °C, 120 h (0.928, 0.898, 0.917, respectively; *p* < 0.01), CRP–20 °C, 72 h (0.935, 0.912, 0.909, respectively; *p* < 0.01), PRCRP–20 °C, 72 h (0.938, 0.934, 0.909, respectively; *p* < 0.01), GFC (0.958, 0.887, 0.879, respectively; *p* < 0.01), CT (0.873, 0.879, 0.890, respectively; *p* < 0.01), AAT (0.847, 0.857, 0.875, respectively; *p* < 0.01), PRET–13 °C, 144 h (0.917, 0.837, 0.855, respectively; *p* < 0.01), PRET–20 °C, 66 h (0.940, 0.888, 0.862, respectively; *p* < 0.01) with FSEs (FSE–J, FSE–L, and FSE–S) (Table 3). In addition, there were significant positive correlations between the four seed vigour indices of RT and other seed vigour indices, and the highest correlation coefficients were for CRP–15 °C, 120 h (0.941), PRCRP–15 °C, 120 h (0.920), CRP–20 °C, 72 h (0.925), and PRCRP–20 °C, 72 h (0.958) with the GFC. These findings showed that the four RT indices can be used as indicators of maize seed lot vigour to predict field seedling emergence.

### 2.8. The Pericarp-Testa Limits Maize Seed Germination

**Figure 4:**
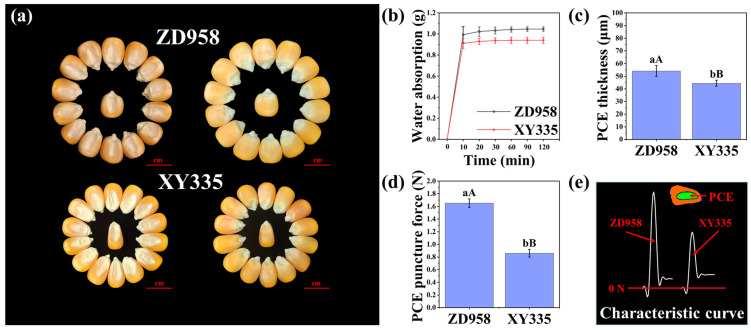
The physical features of seeds from ZD958 (ZhengDan958) and XY335 (XianYu335) are compared. The seed morphology is compared in (**a**); the pericarp–testa water absorption characteristics are compared in (**b**); the pericarp–testa covering embryo (PCE) thickness is compared in (**c**); the PCE puncture force is compared in (**d**); and the puncture force measurement characteristic curve is compared in (**e**).

The principle of the rupture test has been clearly described in this study, but the regulatory mechanism of rupture events needs to be further analysed. Here, the seed lot vigour of ZD958–2021 was significantly lower than that of XY335–2021 (Figure 2 and Table 1), while the Chinese maize cultivar ZD958 and American maize cultivar XY335 are both famous hybrid maize cultivars. Therefore, it is important to compare and analyse the two methods to better understand and apply RT. The seeds of cultivar ZD958 were larger than those of cultivar XY335, and the water absorption characteristics of the pericarp–testa of the two cultivars were similar, showing a trend in rapid water absorption at first and then tending to stabilise (Figure 4). The results showed that the pericarp–testa covering embryo (PCE) of ZD958 (54.12 μm) was significantly greater than that of XY335 (44.43 μm), and the puncture force of ZD958 (1.65 N) was also significantly greater than that of XY335 (0.86 N) (Figure 4). Therefore, the physical characteristics of the PCE may be one of the important reasons for decreasing the speed of germination and increasing the differences between seed lots with different vigour levels. Other mechanisms of the PCE regulating seed germination and vigour need to be further studied.

## 3. Discussion

The distinct spatial and organisational structure of seeds/fruits, which serve as the characteristic dispersion and propagation units of angiosperms and gymnosperms, is crucial for seed germination and the establishment of seedlings [22]. The diploid embryo in the mature seeds of the majority of angiosperms is encased in one or more layers of seed covering. These coverings typically consist of a dead diploid maternal seed coat and a comparatively plentiful living triploid endosperm, both of which are essential for regulating germination [4,23,24,25]. Water absorption triggers the start of seed germination, which is followed by a convoluted sequence of physiological alterations and culminates with the primary root’s (embryonic root) emergence through the surrounding tissue [4,24,26]. Previous studies have shown that the fracture toughness of covering tissue decreases with increasing water content during seed germination, which controls the main mechanical and related characteristics of seed germination, such as the mechanical strength of the seed coat, the weakening of the endosperm, and the potential for embryo growth [5]. The mechanical interaction between various seed tissues and organs is essential for seed development and growth [27]. In particular, the completion of seed germination depends on the balance between the inhibitory force of the seed coat and the growth potential of the hypocotyls. And the occurrence speed of biomechanical events during seed germination is an important manifestation of seed vigour.

For many years, seed vigour testing has been widely used in the seed industry, but it has been difficult to standardise. The seed vigour test can effectively reflect seed performance in the seed deterioration stage. Seed scientists have been searching for a practical, reliable, and standardizable method of seed vigour testing to predict the field seedling emergence performance of seed lots. However, no single vigour test has been performed to assess the vigour of commercially acceptable germinated seeds under standardised conditions. Moreover, the number of standardised seed vigour test methods included by ISTA (International Seed Testing Association) and AOSA (Association of Official Seed Analysts) is still very limited, including conductivity tests, accelerated ageing tests, radicle (primary root) emergence tests, cold tests, etc. Therefore, in order to ensure seed quality and promote the rapid development of the modern seed industry, it is particularly important to develop more new seed vigour detection methods, especially those that can be standardised in the future.

Maize is an important food and forage crop worldwide, and high-vigour seeds have obvious growth advantages and production potential [13]. Two successive processes that take place during *Zea mays* germination are pericarp–testa rupture (PR) and coleorhiza rupture (CR) (Figure 1). In the present work, a new seed vigour test method based on PR and CR for maize seed lots, the rupture test (RT), was proposed. Here, seed lots of eight maize cultivars for three seed production years (2021, 2022, and 2023) were used. Three seed lots of eight different cultivars had different vigour levels, as indicated by the results of the standard germination test, seedling growth test, CT, AAT, and PET. Three seed lots of the same cultivar had vigour levels that were 2023 seed lot > 2022 seed lot > 2021 seed lot, which was consistent with the results of the field seedling emergence test (Table 1, Table 2, and Table S2). The twenty–four hybrid maize seed lots were assessed for vigour using the following metrics in the RT: pericarp–testa rupture percentage (PRP), coleorhiza rupture percentage (CRP), and pericarp–testa rupture + coleorhiza rupture percentage (PRCRP) (Figures S2 and S3 and Table S4). Four germination temperatures (13, 15, 20, and 25 °C) were chosen for this investigation, and four seed germination time ranges (13 °C: 0–192 h; 15 °C: 0–192 h; 20 °C: 0–108 h; 25 °C: 0–168 h) were split into seven time periods, respectively, based on the germination progress curves.

Within a certain range of germination temperatures, high temperatures accelerate the germination speed of maize seed lots [2], but higher temperatures affect the accuracy of germination event statistics; in contrast, low temperatures reduce the germination speed of maize seed lots and increase the difference in vigour level among seed lots [10], but lower temperatures affect the efficiency of vigour evaluation of seed lots via rupture tests. By comparison and analysis, CRP–15 °C for 120 h, PRCRP–15 °C for 120 h, CRP–20 °C for 72 h and PRCRP–20 °C for 72 h were selected for the evaluation of seed vigour. The regressions between the four indices and the FSEs (FSE–J, FSE–L, and FSE–S) were extremely close, and the R^2^ of CRP–15 °C at 120 h had the highest value of 0.90944 (Figures S2 and S3). Therefore, 15 °C and 20 °C were selected as the germination temperatures for RT. CRP–15 °C for 120 h, PRCRP–15 °C for 120 h, CRP-20 °C for 72 h, and PRCRP-20 °C for 72 h were strongly correlated with FSEs (FSE–J, FSE–L, and FSE–S) (*p* < 0.01) (Figure 3). These four indices can be used to distinguish three seed lots with different levels of vigour among eight cultivars (*p* < 0.05) (Table S5). In this study, seed germination biomechanics were combined with the vigour evaluation of the seed lot, and the RT, including the selection and determination of vigour indices, was proposed by using the two successive germination events of PR and CR. The RT results of 24 maize seed lots were consistent with the results of other seed vigour test methods and were strongly significantly (*p* < 0.01) correlated with FSEs (FSE–J, FSE–L, and FSE–S), which indicated that it was effective and feasible to evaluate the vigour of maize seed lots via a rupture test to predict field emergence (Figure 3, Table 3 and Table S4). Hence, the proposed method is robust and effective.

The vigour–related traits of two cultivars, ZD958 (a Chinese cultivar) and XY335 (an American cultivar), whose seed lots were stored for the same time and whose degree of decline in vigour differed, were also compared and analysed from the point of view of seed biomechanics. There were significant differences in the thickness and mechanical resistance of the pericarp–testa–covering embryo (PCE). The seed coat, such as the pericarp-testa, has been shown in numerous studies to be the interface between seeds and the external environment. As such, it can shield the embryo from adverse environmental factors, but it can also mechanically prevent seed germination and may have an impact on gaseous exchanges, which could result in an oxygen shortage in the developing cell [3,4,8,24,28,29,30,31,32,33]. This might be a major factor in the ZD958 seed lot’s delayed germination rate and the larger drop in seed vigour during the same storage period compared to XY335. Overall, the germination speed and vigour of the four Chinese cultivar seed lots were slightly lower than those of the four American cultivar seed lots in the same year, possibly because Chinese maize breeding has long focused on yield, quality, disease resistance, and other traits in the late growth stage but has not paid enough attention to seed vigour traits in the past. As a result, some varieties have genetic defects in terms of seed germination and vigour, including slow germination and poor storage. The United States and China are the two largest maize–producing countries, but the number of seed enterprises in China is far greater than that in America (for example, the number of seed enterprises in China in 2020 was as high as 7372) [34,35]. In particular, there is still a large gap in the quality standards of qualified commercial maize hybrids. For example, the standard germination percentage (SGP) of the maize seed lot of the pioneer enterprise is ≥95.0%, while the national SGP of the maize seed lot of China is only ≥85.0% (the seed lot for single seed sowing is ≥93.0%) [35] (Figure S4).

## 4. Materials and Methods

### 4.1. Seed Lots

Seeds of eight hybrid maize cultivars, ‘DengHai605’, ‘LuDan818’, ‘LongPing206’, ‘ZhengDan958’, ‘DiKa517’, ‘XianYu508’, ‘XianYu047’, and ‘XianYu335’ (coded as DH605, LD818, LP206, ZD958, DK517, XY508, XY047, and XY335, respectively), were used in this study (Table S1). The first four hybrid maize cultivars are Chinese, and the last four are American. Each cultivar had three seed lots, which were purchased in 2021, 2022, and 2023. After use, all the seed samples were placed in the seed bank (10 °C and 40% RH).

### 4.2. Seed Size, Seed Weight, and Seed Water Content

Seed images were obtained by scanning with a scanner (Epson Perfection V800 Photo, Seiko Epson Corporation, Suwa, Japan), and the results were analysed with Seed Identification software in three replicates of 50 seeds each [36]. Take 500 seeds randomly from each seed lot to determine the weight (three replicates) and convert it into a 1000–seed weight. According to the method introduced by ISTA [37], the seeds were ground and dried at 130 ± 0.5 °C for four hours (h) to ascertain the water content. The percentage of water content was then computed using the fresh weight basis.

### 4.3. Light Microscopy

Seeds were photographed using a stereomicroscope (Olympus SZX16, Japan) with an attached Olympus DP72 cooled digital camera. Image–Pro Express 6.0 (Olympus) was used as the digital imaging software to overview the research applications. Pictures of field seedling emergence were taken with a digital camera (EOS 5D Mark IV, Canon (China) Co., Ltd., Shanghai, China).

### 4.4. Germination

Before being utilised for rolled paper germination, randomly selected pure seeds were surface sterilised in 1% NaClO (*w*/*v*) for ten minutes. They were then thoroughly cleaned three times using distilled water. For the germination test using rolled towels, three replicates of one hundred seeds were set for every seed lot. The sterilised seeds were put on germination paper (Anchor Paper Co., St Paul, MN, USA), with the seed pedicel/primary root (embryonic root) pointing to the bottom of the paper, and the rolled paper towel was put in a self–sealing bag. Then, the self–sealing plastic bags with rolled paper towels were upwardly set in an intelligent artificial climate box (MGC–350HP, Shanghai Yiheng Scientific Instrument Co., Ltd., Shanghai, China) at 25 ± 0.5 °C (12 h of light and 12 h of darkness). The germination first count (GFC) and germination percentage (GP) were estimated on the fourth and seventh days after the test had been set up, respectively [10,38]. After the determination of GP, 10 seedlings/plants of uniform size were randomly selected from each seed lot with three replicates for the measurement of six indices, including shoot/seedling length (SL), shoot/seedling fresh weight and dry weight (SFW and SDW), primary root length (PRL), root fresh weight and dry weight (RFW and RDW) [39]. For SDW and RDW, the plant tissue was dried at 105 ± 0.5 °C for 8 h [39]. The germination index (GI) and vigour index (VI) were determined with the accompanying formula [40]:

GI = ∑(Gt/Dt)(1)

where Gt is the number of germinated seeds at Dt; Dt is the number of days from when germination occurred;

VI = GI × SL(2)

### 4.5. Cold Test (CT), Accelerated Ageing Test (AAT), and Primary Root Emergence Test (PRET)

The CT and AAT were conducted according to the seed quality testing and evaluation manual of corn (*Zea mays*) [41] and ISTA rules [10], respectively. For the CT, seeds were incubated in moist soil media with approximately 70% water–holding capacity taken from a maize field at 10 ± 0.5 °C for seven days and then transferred to 25 ± 0.5 °C for seven days prior to normal seedling development. Each seed lot was repeated three times, with 100 seeds per repetition. For the AAT, each group of 100 seeds with three replicates was placed in a seed artificial ageing test chamber (LH–150S, Qianjiang Instrument & Equipment Co., Ltd., Hangzhou, China) precisely set at 43 ± 0.3 °C and 98% RH for 72 h, followed by a standard germination test at 25 ± 0.5 °C. A rolled paper towel germination test method at 13 ± 0.5 °C and 20 ± 0.5 °C was used for the PRET, and the primary root emergence percentage was expressed as the percentage of plants that produced a primary root ≥ 2 mm long at “13 ± 0.5 °C, 144 ± 1 h” and “20 ± 0.5 °C, 66 h ± 15 min” after sowing [10].

### 4.6. Rupture Test

Based on pericarp–testa rupture (PR) and coleorhiza rupture (CR) during maize seed germination (Figure 1), three seed vigour indices, namely, pericarp–testa rupture percentage (PRP), coleorhiza rupture percentage (CRP), and pericarp–testa rupture + coleorhiza rupture percentage (PRCRP), were evaluated as follows: the PRP (%) = (number of pericarp–testa ruptured seeds/number of seeds tested) × 100; the CRP (%) = (number of coleorhiza ruptured seeds/number of seeds tested) × 100; and the PRCRP (%) = PRP (%) + CRP (%).

With reference to the primary root emergence test (i.e., radicle emergence) [10], four temperatures—13 ± 0.5 °C, 15 ± 0.5 °C, 20 ± 0.5 °C, and 25 ± 0.5 °C—were chosen for the RT. The pure seeds (3 replicates with 100 seeds per replicate) were used for germination on paper towels following the normal procedure for a rolled paper towel germination test. Two rolled paper towels were used for each replicate. The 50 sterilised seeds were placed on germination paper in four rows, with the seed pedicel/primary root (embryonic root) pointing to the bottom of the paper. Seeds in two adjacent rows were staggered. The towels were rolled and placed upright in plastic self–sealing bags for germination. Then, the plastic self-sealing bags with rolled paper towels were vertically put in a plastic basket and then placed in a climate chamber (MGC-350HP, Shanghai Yiheng Scientific Instrument Co., Ltd., Shanghai, China) at the required temperatures. The values of PR and CR were measured and recorded every 24 h ± 1 h from 48 to 192 h at 13 ± 0.5 °C and 15 ± 0.5 °C, every 12 h ± 1 h from 36 to 108 h at 20 ± 0.5 °C, and every 24 h ± 1 h from 24 to 168 h at 25 ± 0.5 °C.

### 4.7. Field Seedling Emergence

Twenty–four maize seed lots were planted at Jimo (J) (Shandong Province), Laiyang (L) (Shandong Province), and Shenzhou (S) (Hebei Province), China, in 2023. The row spacing was 0.25 m, and the spacing between rows was 0.6 m (Figure 1). The seeds were planted using the single–seed sowing technique. After 15 days of sowing, the number of normal seedlings was counted, and the field seedling emergence (FSE) percentage was calculated.

### 4.8. Pericarp–Testa Characteristic Measurements

(i) Water absorption measurements: Seeds were soaked in warm water (30–35 °C) for 4–8 h, and then the pericarp–testa was separated and dried at room temperature to a constant weight for later use, with three replicates of 50 seeds each. At 25 ± 0.5 °C, the samples were gently wiped free of surface water to measure their weight, and the water content was calculated after the samples had absorbed water for 10, 20, 30, 60, 90, and 120 min. (ii) Pericarp–testa thickness measurements: After imbibition for 24 h, the seeds were cut longitudinally along the middle part and sliced. The pericarp–testa covering embryo (PCE) thickness was measured at multiple points (>3) with a Leica DM6B microscope and image analysis software, with three replicates of 20 seeds each. The thickness of the PCE was expressed as the average of the three values in the middle of the dataset. (iii) Puncture force measurements: The self-developed biomechanical evaluation system (A System for Automated Seed Biomechanical Assessment, ASASBA) with a 0.5 mm diameter metal probe was used to measure the PCE puncture force [11]. After imbibition for 24 h, the PCE was separated from the seed with a scalpel and tweezers, and the sample was fixed on a specific mould with three replicates of 30 samples each. In the measurement of sample puncture force, the downward moving speed of the metal probe was 30 mm·min^–1^, and the puncture force data information was recorded in real time. The puncture force of the PCE was expressed as the average of 6 values in the middle of the dataset.

### 4.9. Data Analysis

The data were compared and statistically analysed by the SPSS 11.0 and SAS statistical software (version 9.3) including Fisher’s LSD test, etc. [42]. All the experiments were completed from March to September 2023.

## 5. Conclusions

As a result, data from both vigour indices (PRCRP and CRP) at the same germination temperature can be obtained simultaneously. This is particularly useful when assessing the vigour of a maize seed lot with a slow germination speed since the evaluation result will be more accurate when the two indices at the same germination temperature are used together. Finally, the evaluation system for maize seed lot vigour, the rupture test method, was as follows (Figure 3):

(i) At 15 ± 0.5 °C, the recommended indices are ‘CRP, 120 h ± 1 h’ and ‘PRCRP, 120 h ± 1 h’. For example, if the test is set up at 10:00 a.m., the count will occur at 10:00 a.m. five days later (count at 5 days ± 1 h).
(ii) At 20 ± 0.5 °C, the recommended indices are ‘CRP, 72 h ± 15 min’ and ‘PRCRP, 72 h ± 15 min’. For example, if the test is set up at 10:00 a.m., the count will occur at 10:00 a.m. three days later (count at 3 days ± 15 min).

The ability of the seed embryo to continue its metabolic activities in a coordinated and sequential way during germination is what drives germination vigour. Thus, more research is needed to determine the molecular–regulating mechanisms of PR and CR in maize seed germination. In this case, the rupture test—a suggested method—is effective and provides an important reference for more crops to select for seed germination event(s) and establish corresponding new methods for seed vigour tests in the future.

## Figures and Tables

**Table 1:** Results of the cold test (CT), accelerated ageing test (AAT), and primary root emergence test (PRET) of 24 maize seed lots.

Seed Lots	CT (%)	AAT (%)	PRET–13 °C, 144 h (%)	PRET–20 °C, 66 h (%)
DH605–2023	93 aA	85.33 aA	75.67 aA	89.67 aA
DH605–2022	90 bAB	80.33 bA	72 bB	88 bA
DH605–2021	87 cB	72 cB	65.33 cC	85.67 cB
LD818–2023	92 aA	85 aA	76.67 aA	88.33 aA
LD818–2022	88 bB	79 bB	72.33 bB	86.67 aAB
LD818–2021	85.33 cB	74 cC	69.67 cB	84.33 bB
LP206–2023	91.67 aA	83.67 aA	73.67 aA	88.33 aA
LP206–2022	88 bAB	77.33 bAB	70.67 bAB	85 bAB
LP206–2021	84 cB	70.33 cB	67.33 cB	81.33 cB
ZD958–2023	93.67 aA	85.67 aA	71.33 aA	89.33 aA
ZD958–2022	88.67 bB	75.67 bB	68.33 abAB	85.67 bB
ZD958–2021	84 cC	70.33 cB	65.33 bB	80.67 cC
DK517–2023	90.67 aA	83.33 aA	75.67 aA	88.33 aA
DK517–2022	87 bB	74.67 bAB	72 bB	86.67 abA
DK517–2021	84.67 cB	67.67 cB	67.67 cC	85.67 bA
XY508–2023	91.67 aA	86.67 aA	78 aA	90.67 aA
XY508–2022	88.67 bAB	77 bB	74 bAB	88 bB
XY508–2021	85.33 cB	71.67 cB	71 cB	86.33 cB
XY047–2023	91.67 aA	86 aA	79.33 aA	92 aA
XY047–2022	88 bB	78 bB	75 bAB	89 bB
XY047–2021	85.67 cC	70 cC	72.33 bB	86.67 cB
XY335–2023	93.33 aA	87.67 aA	79 aA	93 aA
XY335–2022	91 bAB	81.67 bA	75 bAB	90.67 bAB
XY335–2021	88 cB	71 cB	72.67 cB	88.33 cB

For the complete list of abbreviations, go to “Figure S1”. The analysis of variance was the same as above.

**Table 2:** Results of the field seedling emergence of 24 maize seed lots.

Seed Lots	FSE–J	FSE–L	FSE–S
DH605–2023	91.33 aA	89.67 aA	90 aA
DH605–2022	87 bB	86.67 bAB	86 bAB
DH605–2021	83.33 cC	82 cB	81.67 cB
LD818–2023	90.33 aA	88.33 aA	90 aA
LD818–2022	86.33 bAB	85.67 bA	86.33 bB
LD818–2021	82.33 cB	79.33 cB	83.33 cC
LP206–2023	87.67 aA	87 aA	89.67 aA
LP206–2022	84.33 aAB	85.67 aA	85.67 bB
LP206–2021	80.33 bB	74.33 bB	81.67 cC
ZD958–2023	87.67 aA	90 aA	89.67 aA
ZD958–2022	85.33 bA	86 bA	87 bB
ZD958–2021	78 cB	79.33 cB	79.67 cC
DK517–2023	90.33 aA	91 aA	92.33 aA
DK517–2022	88 aAB	86.67 bAB	88 bAB
DK517–2021	84 bB	80.33 cB	85 bB
XY508–2023	91 aA	90.67 aA	90.67 aA
XY508–2022	87 bB	86.67 bB	87.33 bAB
XY508–2021	83.33 cC	82 cC	84 cB
XY047–2023	92 aA	92 aA	91 aA
XY047–2022	87.67 bB	89.67 bB	86.67 bAB
XY047–2021	85.33 cC	83.67 cC	82.67 cB
XY335–2023	93.33 aA	92 aA	92.67 aA
XY335–2022	89.67 bAB	88 bAB	90 bAB
XY335–2021	86 cB	84.33 cB	86.33 cB

The indices evaluated were field seedling emergence (FSE) at the Jimo experimental station (FSE–J), FSE at the Laiyang experimental station (FSE–L), and FSE at the Shenzhou experimental station (FSE–S). The analysis of variance was the same as above.

**Table 3:** Correlation analysis between maize seed vigour indices and FSEs.

Indices	A	B	C	D	E	F	G	H	I	J	K
**B**	0.994 **										
**C**	0.913 **	0.883 **									
**D**	0.919 **	0.890 **	0.988 **								
**E**	0.927 **	0.898 **	0.932 **	0.941 **							
**F**	0.910 **	0.918 **	0.801 **	0.829 **	0.817 **						
**G**	0.901 **	0.916 **	0.733 **	0.764 **	0.776 **	0.946 **					
**H**	0.879 **	0.859 **	0.853 **	0.866 **	0.918 **	0.754 **	0.799 **				
**I**	0.905 **	0.877 **	0.916 **	0.927 **	0.948 **	0.839 **	0.777 **	0.879 **			
**J**	0.956 **	0.928 **	0.935 **	0.938 **	0.958 **	0.873 **	0.847 **	0.917 **	0.940 **		
**K**	0.921 **	0.898 **	0.912 **	0.934 **	0.887 **	0.879 **	0.857 **	0.837 **	0.888 **	0.917 **	
**L**	0.933 **	0.917 **	0.909 **	0.909 **	0.879 **	0.890 **	0.875 **	0.855 **	0.862 **	0.940 **	0.891 **

A: CRP–15 °C, 120 h, B: PRCRP–15 °C, 120 h, C: CRP–20 °C, 72 h, D: PRCRP–20 °C, 72 h, E: GFC, F: CT, G: AAT, H: PRET–13 °C, 144 h, I: PRET–20 °C, 66 h, J: FSE–J, K: FSE–L, L: FSE–S. For the complete list of abbreviations, go to “Figure S1”. ** indicates significance at *p* < 0.01.

## Data Availability

The original contributions presented in the study are included in the article/Supplementary Materials, further inquiries can be directed to the corresponding author.

## Supplementary Materials

The following supporting information can be downloaded at: https://www.mdpi.com/article/10.3390/plants13131847/s1, Figure S1: Abbreviations and full names; Figure S2: Rupture test of 24 maize seed lots at 13 and 15 °C; Figure S3: Rupture test of 24 maize seed lots at 20 and 25 °C; Figure S4: Regression analysis of four seed vigour indices (‘CRP-13 °C, 120 h’, ‘PRCRP-13 °C, 120 h’, ‘CRP-15 °C, 120 h’, ‘PRCRP-15 °C, 120 h’) and FSEs (FSE-J, FSE-L and FSE-S). For the complete list of abbreviations, go to “Figure S1”; Figure S5: Regression analysis of four seed vigour indices (‘CRP-20 °C, 72 h’, ‘PRCRP-20 °C, 72 h’, ‘CRP-25 °C, 72 h’, ‘PRCRP-25 °C, 72 h’) and FSEs (FSE-J, FSE-L and FSE-S). For the complete list of abbreviations, go to “Figure S1”; Figure S6: Comparison of maize seed quality standards and related factors between America and China; Table S1: Maize sample seed information; Table S2: Results of the seed length, seed width, 1000-seed weight, and seed water content of 24 maize seed lots; Table S3: Results of the germination test and seedling growth test of 24 maize seed lots; Table S4: Correlation analysis of the PRP, CRP, and PRCRP with FSEs; Table S5: Results of four rupture test indices for 24 maize seed lots.
