# Supplementary material for: Rupture Test: A New Method for Evaluating Maize (Zea mays) Seed Vigour
Source: Plants (Basel). 2024 Jul 4;13(13):1847. doi: 10.3390/plants13131847 (PMC11243803; doi:10.3390/plants13131847)
Supplement: Supplementary file 1 [file plants-13-01847-s001.zip › plants-3047603-supplementary.pdf]

Abbreviations	Full names
AAT	accelerated aging test
CR	coleorhiza rupture
CRP	coleorhiza rupture percentage
CT	cold test
FSE	field seedling emergence
GFC	germination first count
GI	germination index
GP	germination percentage
SWC	seed water content
PCE	pericarp-testa covering embryo
PET	primary-root emergence test
PR	pericarp-testa rupture
PRL	primary root length
PRP	pericarp-testa rupture percentage
PRCRP	pericarp-testa rupture + coleorhiza rupture percentage
RDW	root dry weight
RFW	root fresh weight
SDW	shoot/seedling dry weight
SFW	shoot/seedling fresh weight
SL	seed length or shoot/seedling length
SW	seed width
TSW	thousand-seed weight
VI	vigour index
10 S	10 seedlings

Figure S1. Abbreviations and full names.

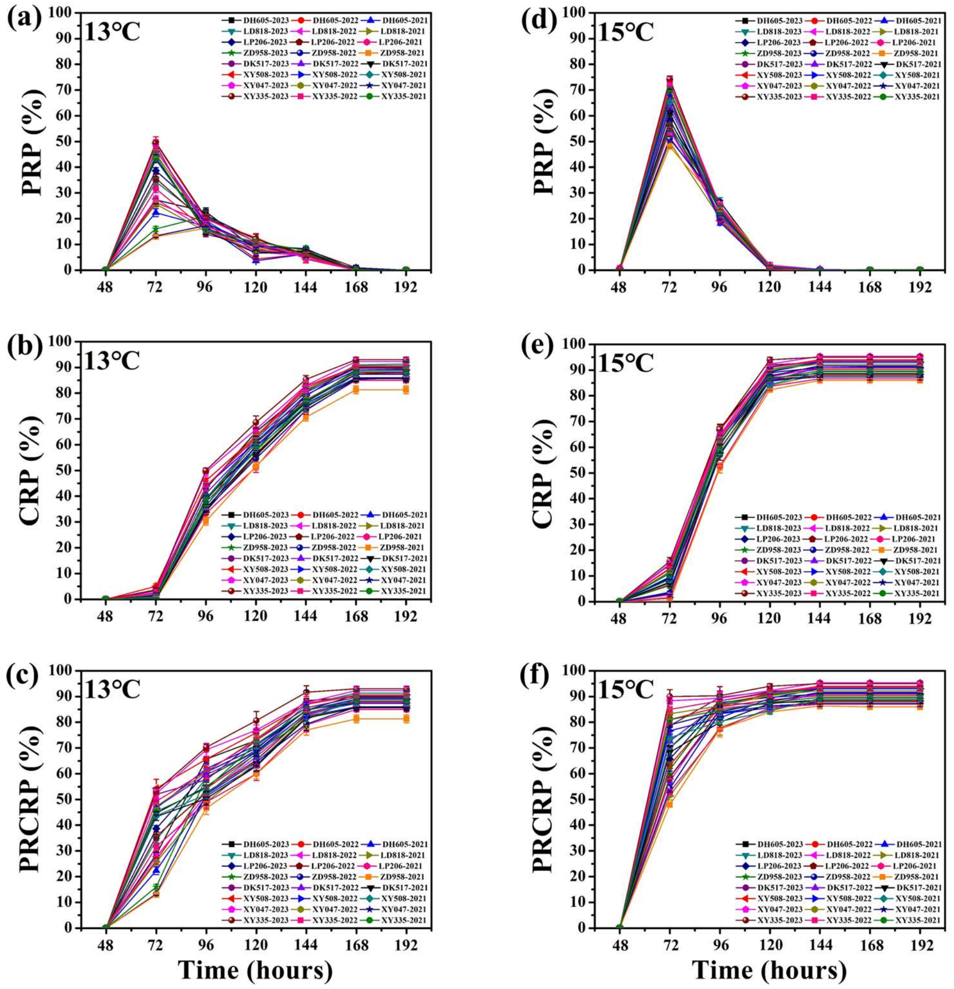

Figure S2. Rupture test of 24 maize seed lots at 13 and 15℃. (**a**) PRP, (**b**) CRP, and (**c**) PRCRP were recorded every 24 h at 13℃; (**d**) PRP, (**e**) CRP, and (**f**) PRCRP were recorded every 24 h at 15℃. For the complete list of abbreviations, go to "Figure S1".

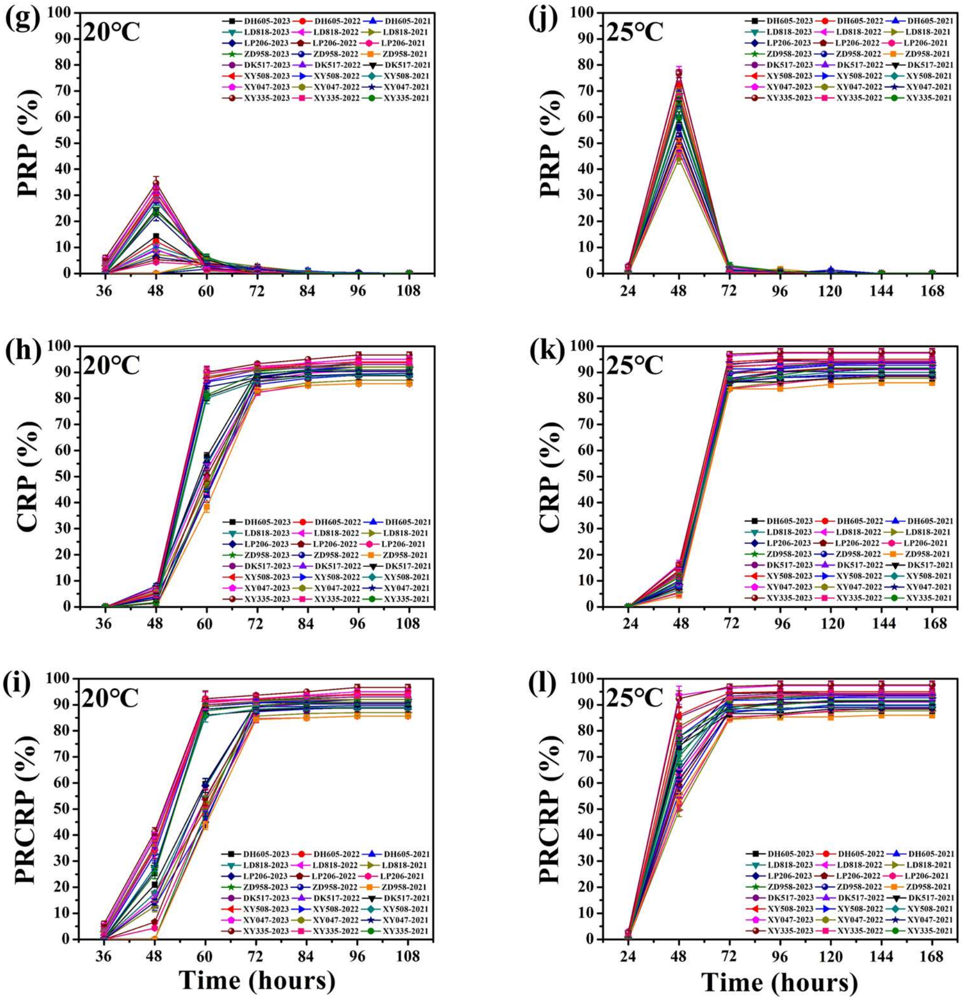

Figure S3. Rupture test of 24 maize seed lots at 20 and 25℃. (**g**) PRP, (**h**) CRP, and (**i**) PRCRP were recorded every 12 h at 20℃; (**j**) PRP, (**k**) CRP, and (**l**) PRCRP were recorded every 12 h at 25℃. For the complete list of abbreviations, go to "Figure S1".

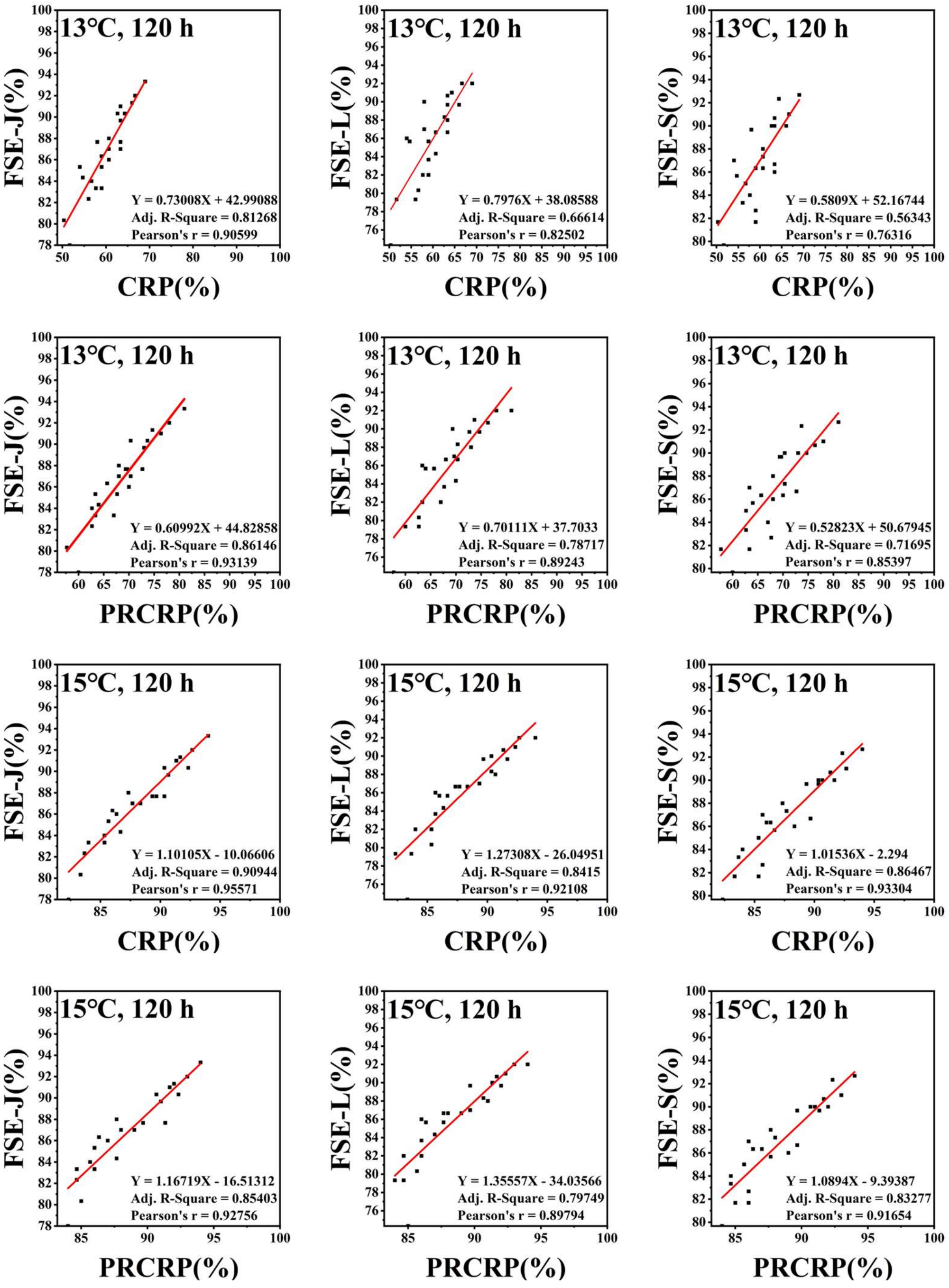

Figure S4. Regression analysis of four seed vigour indices ('CRP-13℃, 120 h', 'PRCRP-13℃, 120 h', 'CRP-15℃, 120 h', 'PRCRP-15℃, 120 h') and FSEs (FSE-J, FSE-L and FSE-S). For the complete list of abbreviations, go to "Figure S1".

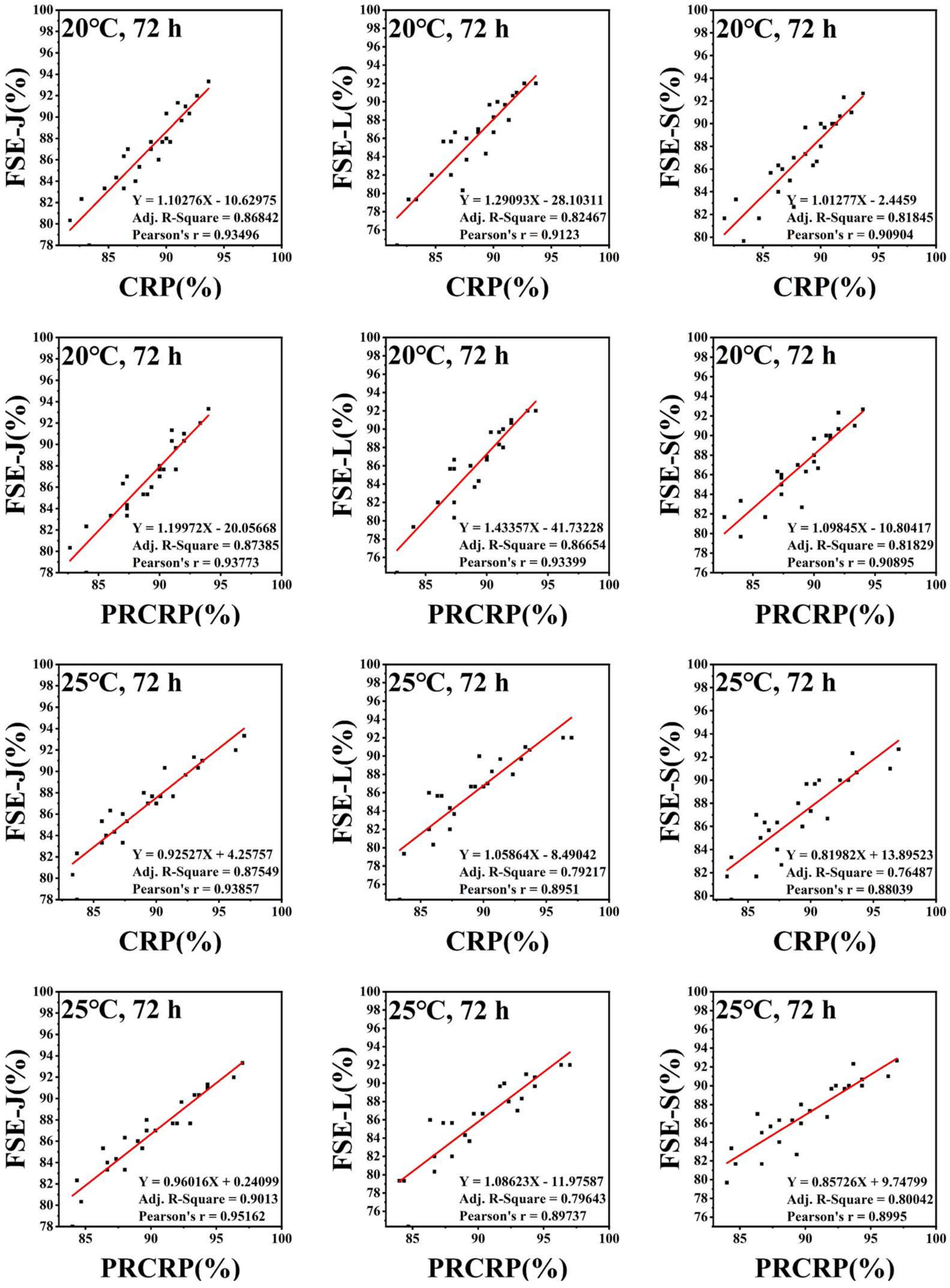

Figure S5. Regression analysis of four seed vigour indices (‘CRP-20℃, 72 h’, ‘PRCRP-20℃, 72 h’, ‘CRP-25℃, 72 h’, ‘PRCRP-25℃, 72 h’) and FSEs (FSE-J, FSE-L and FSE-S). For the complete list of abbreviations, go to "Figure S1".

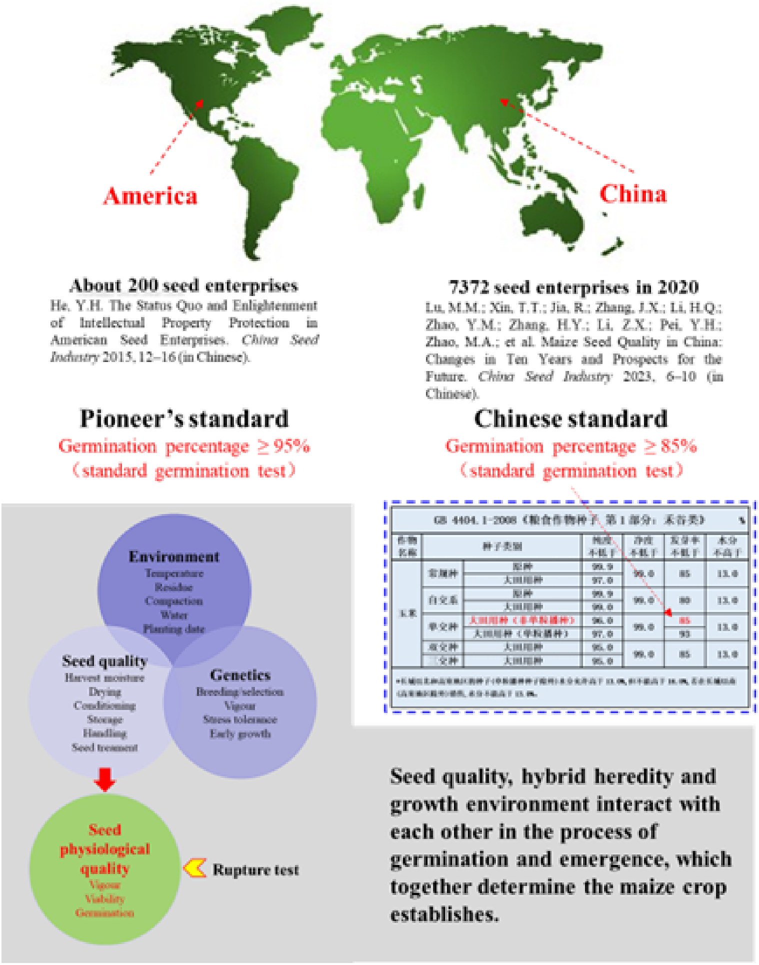

Figure S6. Comparison of maize seed quality standards and related factors between America and China.

Table S1. Maize sample seed information.

Cultivars	♀×♂	Seed production enterprises
DengHai605	DH351×DH382	Shandong Denghai Seed Industry Co., Ltd.
LuDan818	Qx508×Qxh0121	Hefei Fengle Seed Co., Ltd.
LongPing206	L239×L7221	Anhui Longping Hi-Tech Seed Industry Co., Ltd.
ZhengDan958	Zheng58×Chang7-2	Denong Seed Industry Co., Ltd.
DiKa517	D1798Z×HCL645	China Seed International Seed Co., Ltd.
XianYu508	PH6WC×PH5AD	Shandong Denghai Pioneer Seed Industry Co., Ltd.
XianYu047	PH1DP2×PHRKB	Shandong Denghai Pioneer Seed Industry Co., Ltd.
XianYu335	PH6WC×PH4CV	Shandong Denghai Pioneer Seed Industry Co., Ltd.

Table S2. Results of the seed length (SL), seed width (SW), 1000-seed weight (TSW) and seed water content (SWC) of 24 maize seed lots.

Seed lots	SL (mm)	SW (mm)	TSW (g)	SWC (%)	Seed lots	SL (mm)	SW (mm)	TSW (g)	SWC (%)
DH605-2023	11.58aA	9.61aA	346.15aA	11.36aA	DK517-2023	10.83aA	8.78aA	311.27aA	11.46aA
DH605-2022	11.83aA	9.63aA	342.99aA	11.35aA	DK517-2022	10.85aA	8.89aA	311.75aA	11.45aA
DH605-2021	11.86aA	9.71aA	344.61aA	11.31aA	DK517-2021	10.86aA	8.77aA	315.36aA	11.55aA
LD818-2023	11.59aA	9.80aA	362.39aA	11.72aA	XY508-2023	10.75aA	9.02aA	324.12aA	11.34aA
LD818-2022	11.62aA	9.69aA	368.35aA	11.63aA	XY508-2022	10.84aA	8.90aA	322.04aA	11.39aA
LD818-2021	11.50aA	9.62aA	369.18aA	11.7aA	XY508-2021	10.79aA	8.75aA	328.17aA	11.36aA
LP206-2023	11.72aA	9.65aA	364.53aA	11.56aA	XY047-2023	10.57aA	8.61aA	313.74aA	11.32aA
LP206-2022	11.57aA	9.75aA	367.28aA	11.51aA	XY047-2022	10.65aA	8.60aA	319.47aA	11.36aA
LP206-2021	11.63aA	9.61aA	371.31aA	11.54aA	XY047-2021	10.71aA	8.59aA	318.06aA	11.41aA
ZD958-2023	11.81aA	9.50aA	382.92aA	11.47aA	XY335-2023	10.61aA	7.95aA	303.51aA	11.32aA
ZD958-2022	11.58aA	9.57aA	386.09aA	11.56aA	XY335-2022	10.71aA	7.87aA	297.62aA	11.33aA
ZD958-2021	12.14aA	9.66aA	382.54aA	11.51aA	XY335-2021	10.64aA	7.89aA	295.81aA	11.35aA

Different lowercase letters after the values of different seed lots of the same cultivar indicate significant differences at the level of $P < 0.05$, and different uppercase letters indicate extremely significant differences at the level of $P < 0.01$.

Table S3. Results of the germination test and seedling growth test of 24 maize seed lots.

Seed lots	GFC (%)	GP (%)	SL (cm)	PRL (cm)	SFW (g/10 S)	RFW (g/10 S)	SDW (g/10 S)	RDW (g/10 S)	GI	VI
DH605-2023	95.67aA	96.67aA	10.03aA	13.54aA	4.05aA	2.19aA	0.37aA	0.19aA	33.78aA	338.97aA
DH605-2022	91.67bB	94aA	9.65bA	12.88bA	3.85bAB	2.15abA	0.34aAB	0.17bAB	32.4bB	312.65bB
DH605-2021	87.33cC	89.33bB	9cB	11.99cB	3.64cB	2.08bA	0.29bB	0.16bB	30.57cC	275.01cC
LD818-2023	94.33aA	95.33aA	9.98aA	13.78aA	4.02aA	2.27aA	0.36aA	0.21aA	32.48aA	324.02aA
LD818-2022	90.67bA	94aA	9.37bB	12.81bAB	3.89abA	2.11bB	0.34abA	0.17bB	31.7bA	297.14bB
LD818-2021	85cB	90.33bB	8.67cC	11.55cB	3.7bA	2.04bB	0.3bA	0.16bB	30.02cB	259.52cC
LP206-2023	93aA	94.33aA	9.47aA	11.64aA	3.85aA	2.17aA	0.36aA	0.19aA	32.22aA	305.26aA
LP206-2022	88.33bB	93aAB	9.27aAB	11.09bA	3.64bA	1.94bB	0.32bAB	0.15bB	31.26bB	289.76bA
LP206-2021	85.33cB	89.33bB	8.7bB	10.96bA	3.65bA	1.89bB	0.27cB	0.13bB	29.76cC	258.71cB
ZD958-2023	94aA	96aA	9.63aA	12.56aA	3.82aA	2.34aA	0.33aA	0.21aA	32.62aA	314.30aA
ZD958-2022	87.33bB	92.33bA	9.15abAB	12.03bB	3.63bAB	2.12bB	0.31aAB	0.18bAB	31.28bB	286.25bA
ZD958-2021	82cC	87.33cB	8.63bB	11.08cC	3.56bB	1.88cC	0.26bB	0.15bB	29.07cC	250.74cB
DK517-2023	95aA	96aA	10.41aA	13.12aA	3.91aA	2.19aA	0.35aA	0.19aA	33.8aA	351.83aA
DK517-2022	92.33aAB	94.33abA	9.78bA	12.22bAB	3.78bB	2.14aAB	0.33bAB	0.18aA	32.53bA	318.19bB
DK517-2021	88.67bB	92bA	9.02cB	11.37cB	3.69cB	1.99bB	0.3cB	0.15bB	31.07cC	280.20cC
XY508-2023	95.33aA	96aA	11.01aA	12.31aA	3.9aA	2.17aA	0.35aA	0.19aA	34.12aA	375.54aA
XY508-2022	93abAB	94.67abA	10bB	12.12aA	3.75abA	2.08bAB	0.31bAB	0.17bAB	32.67bB	326.62bB
XY508-2021	90bB	92.33bA	9.27cB	11.31bB	3.71bA	2.03bB	0.3bB	0.16cB	30.89cC	286.32cC
XY047-2023	96.67aA	97.33aA	11.55aA	14.28aA	4.18aA	2.33aA	0.37aA	0.2aA	34.9aA	403.24aA
XY047-2022	94.33abA	96abA	10.56bB	13.5bB	4.11aAB	2.19bB	0.34abA	0.19abA	33.68bAB	355.52bB
XY047-2021	92.33bA	93.67bA	9.83cC	12.77cC	3.94bB	2.19bB	0.31bA	0.17bA	32.56cB	320.05cC
XY335-2023	98.33aA	98.33aA	11.2aA	14.5aA	4.35aA	2.36aA	0.37aA	0.22aA	35.22aA	394.56aA
XY335-2022	94.67bAB	95.33bAB	10.72bA	13.28bB	4.22aAB	2.23bB	0.35abAB	0.2bAB	33.65bB	360.85bB
XY335-2021	91.33cB	93bB	9.86cB	12.62cB	3.86bB	2.18bB	0.34bB	0.17cB	32.01cC	315.72cC

Germination first count (GFC), germination percentage (GP), shoot/seedling length (SL), primary root length (PRL), shoot/seedling fresh weight (SFW), root fresh weight (RFW), shoot/seedling dry weight (SDW), root dry weight (RDW), germination index (GI), and vigour index (VI) were the indices that were assessed. 10 S stands for 10 seedlings. The analysis of variance was the same as above.

Table S4. Correlation analysis of the PRP, CRP, and PRCRP with FSEs.

13°C	**48 h**	**72 h**	**96 h**	**120 h**	**144 h**	**168 h**	**192 h**	
FSE-J	0.000	0.529**	0.581**	0.382	-0.339	-0.089	0.000	
FSE-L	0.000	0.374	0.592**	0.431*	-0.36	0.076	0.000	PRP
FSE-S	0.000	0.466*	0.546**	0.499*	-0.443*	0.020	0.000	
FSE-J	0.000	0.662**	0.889**	0.895**	0.922**	0.932**	0.932**	
FSE-L	0.000	0.617**	0.804**	0.814**	0.809**	0.843**	0.857**	CRP
FSE-S	0.000	0.605**	0.812**	0.742**	0.823**	0.872**	0.881**	
FSE-J	0.000	0.561**	0.911**	0.907**	0.874**	0.932**	0.932**	
FSE-L	0.000	0.413*	0.847**	0.861**	0.746**	0.857**	0.857**	PRCRP
FSE-S	0.000	0.496*	0.836**	0.831**	0.734**	0.881**	0.881**	
**15°C**	**48 h**	**72 h**	**96 h**	**120 h**	**144 h**	**168 h**	**192 h**	
FSE-J	0.582**	0.663**	-0.143	-0.533**	-0.473*	0.000	0.000	
FSE-L	0.507*	0.577**	0.01	-0.532**	-0.492*	0.000	0.000	PRP
FSE-S	0.526**	0.531**	0.028	-0.413*	-0.418*	0.000	0.000	
FSE-J	0.000	0.846**	0.912**	0.945**	0.927**	0.929**	0.929**	
FSE-L	0.000	0.751**	0.837**	0.892**	0.860**	0.860**	0.860**	CRP
FSE-S	0.000	0.771**	0.781**	0.894**	0.843**	0.844**	0.844**	
FSE-J	0.000	0.746**	0.908**	0.929**	0.924**	0.927**	0.927**	
FSE-L	0.000	0.654**	0.916**	0.871**	0.854**	0.854**	0.854**	PRCRP
FSE-S	0.000	0.631**	0.866**	0.898**	0.841**	0.843**	0.843**	
**20°C**	**36 h**	**48 h**	**60 h**	**72 h**	**84 h**	**96 h**	**108 h**	
FSE-J	0.650**	0.571**	-0.522**	-0.483*	-0.148	-0.019	0.000	
FSE-L	0.623**	0.457*	-0.572**	-0.467*	-0.061	0.145	0.000	PRP
FSE-S	0.609**	0.468*	-0.478*	-0.476*	-0.095	0.118	0.000	
FSE-J	0.000	0.688**	0.553**	0.912**	0.952**	0.960**	0.960**	
FSE-L	0.000	0.557**	0.458*	0.898**	0.922**	0.932**	0.935**	CRP
FSE-S	0.000	0.570**	0.475*	0.872**	0.883**	0.908**	0.911**	
FSE-J	0.650**	0.654**	0.514*	0.943**	0.953**	0.957**	0.957**	
FSE-L	0.623**	0.526**	0.417*	0.931**	0.933**	0.935**	0.935**	PRCRP
FSE-S	0.609**	0.538**	0.440*	0.896**	0.890**	0.910**	0.910**	
**25°C**	**24 h**	**48 h**	**72 h**	**96 h**	**120 h**	**144 h**	**168 h**	
FSE-J	0.630**	0.714**	-0.125	-0.349	-0.262	0.000	0.000	
FSE-L	0.586**	0.673**	-0.128	-0.218	-0.309	0.000	0.000	PRP
FSE-S	0.604**	0.600**	-0.068	-0.239	-0.428*	0.000	0.000	
FSE-J	0.000	0.866**	0.925**	0.955**	0.950**	0.948**	0.948**	
FSE-L	0.000	0.810**	0.888**	0.919**	0.916**	0.898**	0.898**	CRP
FSE-S	0.000	0.775**	0.856**	0.888**	0.909**	0.893**	0.893**	
FSE-J	0.630**	0.784**	0.956**	0.952**	0.952**	0.948**	0.948**	
FSE-L	0.586**	0.737**	0.915**	0.932**	0.914**	0.897**	0.897**	PRCRP
FSE-S	0.604**	0.717**	0.897**	0.897**	0.892**	0.887**	0.887**	

*, ** indicate significance at $P < 0.05$ and $P < 0.01$, respectively.

Table S5. Results of four rupture test indices for 24 maize seed lots.

Seed lots	CRP-15℃, 120 h (%)	PRCRP-15℃, 120 h (%)	CRP-20℃, 72 h (%)	PRCRP-20℃, 72 h (%)
DH605-2023	91.67aA	92aA	91aA	91aA
DH605-2022	88.33bB	89bAB	86.67bB	87.33bB
DH605-2021	85.33cC	86cB	84.67cB	86cB
LD818-2023	90.33aA	90.67aA	90aA	91aA
LD818-2022	86bB	86.33bB	86.33bB	87bB
LD818-2021	83.67cB	84.67cB	82.67cC	84cB
LP206-2023	89.33aA	89.67aA	88.67aA	90aA
LP206-2022	86.67bA	87.67bAB	85.67bB	87.33bA
LP206-2021	83.33cB	85cB	81.67cC	82.67cB
ZD958-2023	90.33aA	91.33aA	90.33aA	91.33aA
ZD958-2022	85.67bB	86bB	87.67bB	88.67bB
ZD958-2021	82.33cC	84cB	83.33cC	84cC
DK517-2023	92.33aA	92.33aA	92aA	92aA
DK517-2022	87.33bB	87.67bB	90bA	90bB
DK517-2021	85.33cC	85.67cC	87.33cB	87.33cC
XY508-2023	91.33aA	91.67aA	91.67aA	92aA
XY508-2022	87.67bB	88bB	88.67bB	90bA
XY508-2021	84cC	84.67cC	86.33cC	87.33cB
XY047-2023	92.67aA	93aA	92.67aA	93.33aA
XY047-2022	89.67bB	89.67bB	89.67bB	90.33bB
XY047-2021	85.67cC	86cC	87.67cC	89cB
XY335-2023	94aA	94aA	93.67aA	94aA
XY335-2022	90.67bB	91bA	91.33bB	91.33bB
XY335-2021	86.33cC	87cB	89.33cC	89.33cB

The indices evaluated were the CRP at 15℃ for 120 h (CRP-15℃, 120 h), PRCRP at 15℃ for 120 h (PRCRP-15℃, 120 h), pericarp-testa rupture percentage at 20℃ for 72 h (CRP-20℃, 72 h), and PRCRP at 20℃ for 72 h (PRCRP-20℃, 72 h). The analysis of variance was the same as above.
